# Application of innovative gas-permeable four-layered film for packaging diced radish (*Raphanus sativus* L.) kimchi to extend shelf life under fluctuating temperature conditions

**DOI:** 10.1016/j.fochx.2025.103008

**Published:** 2025-09-08

**Authors:** So Yoon Park, Suk-Min Yun, Jeong-Yong Cho, Bo-Sung Shin, Ho Hyun Chun

**Affiliations:** a Industrial Intelligence Research Division, World Institute of Kimchi, Gwangju 61755, Republic of Korea; b Department of Integrative Food, Bioscience and Biotechnology, Graduate School of Chonnam National University, Gwangju 61186, Republic of Korea; c Department of Optics and Mechatronics Engineering, Pusan National University, Busan 46241, Republic of Korea

**Keywords:** Radish kimchi, Gas-permeable multilayer film, Quality, Packaging, Prediction model

## Abstract

This study aimed to evaluate the effects of high and low gas-permeable (GP) four-layered film bags laminated with a functional film featuring micro- and nano-foamed structures on the headspace gas composition, packaging volume expansion (PVE), and quality of diced radish kimchi (DRK) stored at dynamic temperatures. After 30 days of storage, the volume expansion of high-GP film bags containing DRK was approximately 27 % lower than that of airtight three-layer and low-GP film bags, indicating a lower CO_2_ concentration in the headspace. The different packaging types did not significantly affect the total lactic acid bacteria count, pH, titratable acidity, reducing sugar content, and sample salinity. The Huang model provided an acceptable prediction of the changes in the PVE ratio of DRK stored at dynamic temperatures. Combining high-GP films and predictive PVE ratio models is promising for maintaining kimchi packaging stability and quality during cold chain storage and distribution.

## Highlights

• Gas-permeable film with open/closed pores and porous channels enables CO_2_ release.
• High gas-permeable film bags reduce package swelling compared with the control.
• Huang model accurately predicts acidity and package volume expansion in kimchi.
• Novel gas-permeable films enhance the stability of kimchi packaging during storage.

## 1 Introduction

Since the COVID-19 pandemic, kimchi, a Korean fermented food, has been attracting attention worldwide owing to its various health benefits, including anti-obesity, anti-diabetic, anti-mutagenic, and antioxidant effects. Kimchi has over 200 types, depending on the vegetables used and seasoning mixture added (Park et al., 2012; Surya & Nugroho, 2023). Among these types, kkakdugi, a refreshing kimchi prepared with diced radishes (*Raphanus sativus* L.) without pasteurization, is a staple side dish in Korean cuisine that is enjoyed alongside rice and grilled meat owing to its spicy, tangy flavor and delightful crunch. It also functions as a condiment, adding a burst of sweet and spicy complexity to noodle soups, stews, and fried rice (Surya & Nugroho, 2023).

Naturally existing lactic acid bacteria (LAB) or LAB starters ferment unripened kimchi, resulting in the production of organic acids (lactic, succinic, and acetic acids), ethanol, CO_2_, volatile compounds, and other metabolites. During the cold supply chain, continuous fermentation generates CO_2_, which becomes trapped and accumulates within the headspace of packaging film bags. The accumulation of CO_2_ in kimchi packaging causes package deformation and rupture, resulting in significant financial losses for producers and distributors due to product spoilage and recalls (Lee et al., 2020). Thus, CO_2_ buildup must be eliminated to prevent leakage or packaging rupture caused by distension.

Pouch-type plastic films are the most widely used packaging material in the Korean kimchi industry owing to their affordability compared to plastic trays and rigid containers. They are available in a range of sizes, from 100 g to 10 kg, to meet diverse kimchi packaging needs (Jaisan et al., 2019). Conventional techniques, such as applying a one-way degassing valve or CO_2_ absorbent sachet to plastic film pouches, are employed to release or absorb CO_2_ and prevent kimchi packaging from swelling or bursting due to lactic acid fermentation during cold chain storage and transportation (Yu et al., 2023). Despite their advantages in preserving kimchi packaging, one-way degassing valves significantly raise the price of plastic film bags, posing an economic barrier to their widespread adoption in kimchi packaging. Calcium hydroxide (Ca(OH)_2_), sodium carbonate (Na_2_CO_3_), and magnesium hydroxide (Mg(OH)_2_) are primarily used in fabricating CO_2_ absorbent sachets (Wang et al., 2015). However, integrating a CO_2_ adsorbent into a packaging film increases the complexity and cost of manufacturing. Moreover, if a sachet is damaged, leakage of these compounds within the packaging may pose safety concerns (Lee et al., 2020). In addition, reaching levels close to 0 % oxygen in the headspace of an airtight packaging film with a CO_2_ absorbent sachet may negatively affect the organoleptic quality of kimchi (Lee et al., 2020). Therefore, for kimchi packaging, multi-layer films with enhanced gas permeability to maintain appropriate headspace CO_2_ concentration and packaging volume without the need for degassing valves or CO_2_ adsorbents are increasing in demand. In our previous research, a novel gas-permeable (GP) multilayer film was developed for kimchi packaging (Park et al., 2022). This GP film, which is laminated with special polyethylene (PE) film with nano/micro-foamed structures, can maintain an appropriate CO_2_ concentration by releasing the CO_2_ accumulated in the headspace without leaking the kimchi soup.

Temperature fluctuations during refrigerated transport, showcases at stores, and even household refrigerators can considerably affect the microbial communities in and quality of commercial kimchi products (Song et al., 2020). Specifically, the distribution temperature history may significantly affect the maturity level of these products (Jaisan & Lee, 2017). Kimchi products that lost their commercial value due to long-term exposure to inappropriate distribution temperatures are typically discarded via landfilling or incineration, resulting in economic loss and environmental burden (Lee et al., 2020).

The primary shelf life of foods is the period during which quality indicators are maintained at acceptable levels under specific storage and distribution conditions without opening the packaging (Bianchi et al., 2024). Quality prediction models based on microbiological and physicochemical indicators can quantitatively describe the spoilage potential and determine the primary shelf life of foods (Putnik et al., 2017). Managing quality by monitoring changes in the quality indices of packaged foods in real time in response to actual distribution environment conditions remains difficult for food producers and retailers (Gogou et al., 2015). Therefore, a quality prediction model with guaranteed reliability for the quality control of packaged foods in an actual distribution environment with variable temperature needs to be developed. Quality prediction models using dynamic temperature scenarios consider temperatures that are similar to realistic food storage and distribution conditions (Tsironi et al., 2020). These dynamic prediction models are more useful than those developed using quality data obtained under a certain temperature for describing the changes in quality indices over time (Giannakourou et al., 2005). The optimal edible period and primary shelf life of kimchi depend on its degree of ripening (Kim et al., 2020). However, the ripening quality of kimchi cannot be determined until the packaging is opened. Thus, developing a reliable predictive model for kimchi quality is crucial, especially when considering the dynamic temperature conditions during storage and distribution.

In previous studies, quality prediction models for dairy products (Chaturvedi et al., 2023), pickled vegetables (Xing et al., 2021), and fresh-cut fruits (Tsironi et al., 2017) stored under fluctuating temperature conditions were developed. However, few studies have reported the development of dynamic mathematical models that can predict the kinetic bacterial growth and ripening of kimchi products stored at variable temperatures (Kim et al., 2020). Therefore, this study aimed to investigate the effects of GP multilayer films—developed to address package expansion during simulated storage conditions with temperature fluctuations—on the microbiological and physicochemical qualities, as well as the in-package gas composition, of diced radish kimchi (DRK). Subsequently, the collected data on quality and packaging film volume changes were used to develop mathematical quality prediction models for evaluating the long-term preservation potential of DRK. This study provides novel practical tools for managing kimchi shelf life in the kimchi industry.

## 2 Materials and methods

### 2.1 DRK preparation

Commercially manufactured DRK was obtained from a local processing plant in Gimhae, the Republic of Korea, on the day of the experiment. Briefly, spring radish (*Raphanus sativus* L.) harvested in June 2023 was cut into approximately 2 × 2 × 2 cm^3^ cubes using a commercial vegetable cutting machine, salted in 120 g/L brine (solar salt, Shinan, Republic of Korea) for 2 h, and then washed with cold tap water. After the excess water was drained for 2 h, the salted radish cubes were mixed with a red pepper powder-based seasoning mixture at a weight ratio of 85:15. The seasoning mixture comprised red pepper powder (326 g/kg), salt-fermented shrimp (152 g/kg), glutinous rice paste (106 g/kg), refined sugar (98 g/kg), fermented anchovy juice (76 g/kg), garlic (75 g/kg), kelp stock (61 g/kg), onion (53 g/kg), Welsh onion (30 g/kg), and ginger (23 g/kg).

### 2.2 Sample packaging

**Fig. 1:**
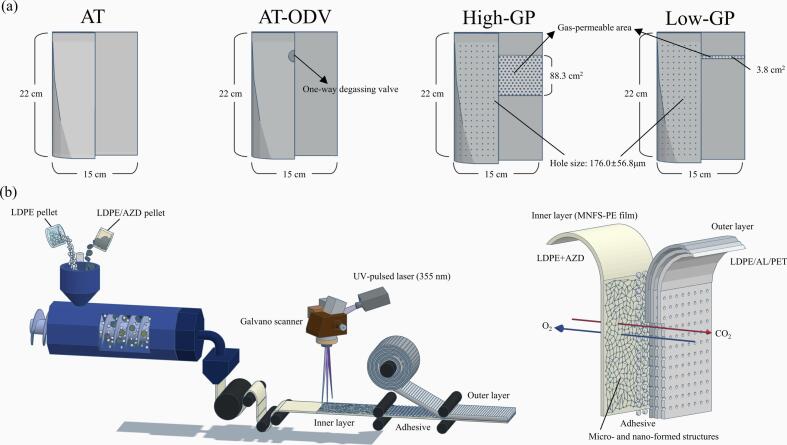
Schematic of the film pouches used for DRK packaging (a) and flow diagram of the fabrication of gas-permeable films (b). DRK, diced radish kimchi.

Fig. 1a shows a schematic of the four types of packaging film pouches used in this study. The airtight three-layer (AT) film pouch was composed of polyethylene terephthalate (PET)/aluminum foil (AL)/low-density polyethylene (LDPE) with a thickness of 0.22 mm and an oxygen transmission rate of 0.05 mL O_2_/m^2^·day·atm. The AT film pouch equipped with a one-way degassing valve was named “AT-ODV.” Two types of GP four-layer film pouches, namely, high and low GP film pouches (high-GP and low-GP, respectively), were fabricated by adhering a micro-perforated (4 holes/cm^2^) three-layer (PET/AL/LDPE) film and a functional LDPE single-layer film (named “MNFS-PE film”), in which micro- or nano-foamed structures were formed using azodicarbonamide (AZD) (Fig. 1b). The MNFS-PE film was produced by combining LDPE pellets and LDPE/AZD pellets in an injection molding machine at approximately 160–180 °C, ensuring a uniform mixture. Subsequently, the film was irradiated with a UV-pulsed laser at a 355 nm wavelength, employing a Galvano scanner for the process. LDPE permits the UV-pulsed laser beam to pass through, while AZD captures its energy. Upon absorbing sufficient laser energy, AZD undergoes dynamic evaporation, releasing N_2_, CO, and CO_2_ gases (Park et al., 2022). This process creates micro- or nano-foamed structures that enhance the gas permeability of the films. The resulting unique structure, featuring open or closed pores and porous channels within the high-GP and low-GP film pouches, facilitates the release of CO_2_. The high-GP film pouch, composed of a functional LDPE single-layer film with an area of  88.3 cm^2^, had a thickness of 0.22 mm and an oxygen transmission rate of 13.5 mL O_2_/m^2^·day·atm. The low-GP film pouch, composed of a functional LDPE film with an area of 3.8 cm^2^, had a thickness of 0.22 mm and an oxygen transmission rate of 0.8 mL O_2_/m^2^·day·atm. O_2_ permeability was determined in accordance with the ASTM D3985–17 standard. Each 300 g DRK sample was placed in the AT (control), AT-ODV, high-GP, and low-GP film pouches.

### 2.3 Storage conditions

DRK samples packaged in the four different types of film were stored under three-stage fluctuating temperature conditions using automatic temperature controller (LCH-11, Jeio Tech, Daejeon, Republic of Korea). The storage temperatures and durations were based on the temperature-controlled regime for kimchi—from manufacturing to retail shelves and ultimately to consumer refrigerators—as reported by Park et al. (2022) and Kim et al. (2021): 0 °C during 0–6 days; 6 °C during 7–14 days; and 4 °C during 15–30 days (Table 1). Each experiment was conducted using 108 packages of DRK after 0, 3, 6, 10, 14, 18, 22, 26, and 30 days of storage (4 packages × 9 time-points × 3 replicates).

**Table 1:** Three-stage fluctuating temperature conditions for the storage experiments of packaged DRK.

Storage stage	Early	Middle	Late
Storage period (day)	0–6	7–14	15–30
Storage temperature (°C)	0	6	4

DRK, diced radish kimchi.

### 2.4 Storage experiments

#### 2.4.1 Headspace gas composition and packaging volume expansion (PVE) rate

The headspace O_2_ and CO_2_ concentrations inside each film pouch containing DRK were analyzed using a portable gas analyzer (CheckPoint 3, AMETEK MOCON, Brooklyn, MN, USA) as previously described by Lee et al. (2021). A septum was affixed to the packaging surface prior to the analysis to ensure measurement accuracy. A syringe needle was inserted in each pouch to extract the gas sample. Headspace O_2_ and CO_2_ concentrations were expressed in kPa.

The packaging volume was determined using the water displacement method with slight modifications (Acerbi et al., 2016). Each film pouch was submerged in a 5 L beaker filled with distilled water until all of the displaced water overflowed outside the beaker. The amount of displaced water was measured and assumed equal to the numerical value of the packaging volume (L). The PVE ratio was expressed as V/V_0_, where V represents the packaging volume on a specific day and V_0_ represents the initial packaging volume (Raiola et al., 2020).

#### 2.4.2 Microbiological analysis

Total lactic acid bacteria (TLAB) and total coliform (TC) counts were determined using the AOAC method. Each DRK sample (10 g) was placed in a sterile Whirl-Pak® bag (Nasco, Fort Atkinson, WI, USA) containing 90 mL of sterile (0.85 %) saline solution (HAPS DW-90, HUKO FS, Seoul, Republic of Korea) in a laminar flow hood. After being homogenized for 3 min using a laboratory stomacher, the DRK and saline mixtures were serially diluted 10-fold with sterile 0.85 % saline solution. For TLAB and TC counts, diluted samples (1 mL) were plated on Petrifilm LAB count plates (3 M Co., St. Paul, MN, USA) and Petrifilm CC count plates (3 M Co.) in triplicate. The LAB and TC count plates were incubated at 30 °C for 48 h and 37 °C for 24 h, respectively. The microbial count was expressed as colony-forming units per gram (CFU/g).

#### 2.4.3 pH, titratable acidity (TA), reducing sugar (RS) content, and salinity

The DRK samples were homogenized using a hand blender to analyze the pH, TA, RS content, and salinity. The pH of the homogenized DRK samples was determined using a pH meter (Orion Versa Star Pro, Thermo Scientific, MA, USA), which was calibrated with pH 4.0 and 7.0 buffer solutions at room temperature (25 °C). To determine TA, the homogenized DRK sample was filtered using a sterile cotton gauze. The filtrate was titrated until it reached pH 8.3 using an automatic titrator (TitroLine 5000, SI Analytics, Mainz, Germany) with a 0.1 N NaOH solution. The TA (g/L) was calculated using the equation described by Lee et al. (2021).

The RS content of the DRK samples was measured using the 3,5-dinitrosalicylic acid (DNS) method as described by Choi et al. (2019) with slight modifications. A mixture comprising 1 mL of diluted DRK sample and 3 mL of DNS reagent was transferred into a test tube and heated in a water bath at 100 °C for 5 min. The reaction solution was cooled in an ice-water bath for 10 min, and the RS content was determined by measuring the absorbance of the reaction solution at a wavelength of 550 nm. The RS content was presented as glucose equivalents (mg/g). The salinity of the homogenized DRK samples was measured at 25 °C using a digital salt meter (PAL-SALT, Atago Co. Ltd., Tokyo, Japan). All experiments were performed in triplicate.

### 2.5 Kinetic modeling of TA and PVE rate changes during storage

Changes in the TA and PVE ratio of the packaged DRK samples under fluctuating storage temperature conditions were predicted using the linear regression, Gompertz, logistic regression, and Huang models following Eqs. (1), (2), (3), (4) (Chevallier et al., 2012; Jaisan & Lee, 2017).

Linear regression model:

(1) $$y\left(d\right)=\mathrm{at}+b$$

Modified Gompertz model:

(2) $$y\left(d\right)=y_{0}+\left(y_{\max}-y_{0}\right)\times \exp\left\{-\exp\left[\frac{\mu_{\max}\times e}{\left(y_{\max}-y_{0}\right)}\times \left(\lambda -d\right)+1\right]\right\}$$

Logistic regression model:

(3) $$y\left(d\right)=y_{0}+\frac{\left(y_{\max}-y_{0}\right)}{1+\exp\left(-\mu_{\max}\times \left(d-M\right)\right)}$$

Huang model:

(4) $$\left\{\begin{array}{c} y\left(d\right)=y_{0}+y_{\max}-\ln\;\left\{\exp\left(y_{0}\right)+\left[\exp\left(y_{\max}\right)-\exp\left(y_{0}\right)\right]\times \exp\left[-\mu_{\max}\times B\left(d\right)\right]\right\}\\ B\left(d\right)=d+\frac{1}{\alpha }\times \ln\frac{1+\exp\left[\alpha \times \left(\lambda -d\right)\right]}{1+\exp\left(\alpha \times \lambda \right)} \end{array}\right\}$$

where *y*_(*d*)_ denotes the TA (g/L) or PVE ratio on a certain storage day (d); *a* is the slope parameter; *b* is a constant; *y*_*0*_ is the initial TA (g/L) or initial PVE ratio; *y*_*max*_ is the maximum TA (g/L) or maximum PVE ratio; *μ*_*max*_ denotes the maximum TA increase rate (g/L d^−1^) or maximum PVE ratio (d^−1^); *λ* is the lag time (d); *M* is the time at which the absolute increase rate is maximum in days; and *B* is the time variable.

### 2.6 Evaluation of the model performance

The performance and accuracy of the predictive models were verified by calculating the adjusted coefficient of determination (*R*^*2*^_*ad*j_), root mean square error (*RMSE*), accuracy factor (*A*_*f*_), bias factor (*B*_*f*_), and corrected Akaike information criterion (*AICc*) using Eqs. (5), (6), (7), (8), (9), respectively.

(5) $$R_{\mathrm{adj}}^{2}=1-\left(\frac{n-1}{n-p}\right)\left(\frac{\mathrm{SSE}}{\mathrm{SST}}\right)$$

(6) $$\mathrm{RMSE}=\sqrt{\frac{1}{n}\;\sum_{i=1}^{n}\left(y_{i}-{\hat{y}}_{i}\right)2}\;$$

(7) $$A_{f}=10^{\frac{\sum \left|\log\left(y_{i}/{\hat{y}}_{i}\right)\right|}{n}}$$

(8) $$B_{f}=10^{\frac{\sum \log\left(y_{i}/{\hat{y}}_{i}\right)}{n}}$$

(9) $$\mathrm{AICc}=n\ln\left(\frac{\mathrm{SSE}}{n}\right)+2\left(p+1\right)+\frac{2\left(p+1\right)\left(p+2\right)}{n-p-2}$$

where *n* refers to the number of observations, *p* denotes the number of parameters of the model, *SSE* and *SST* are the sum of squares of errors and the total sum of squares, respectively, *y*_*i*_ and *ŷ*_*i*_ are the observed and predicted values, respectively, and *ȳ*_*i*_ is the mean real observed value.

### 2.7 Statistical analysis

A one-way analysis of variance (ANOVA) was performed to determine significant differences among groups, and Duncan's multiple range test was carried out as a post-hoc test at a 95 % confidence level using SPSS (SPSS Inc., Chicago, United States). A two-way ANOVA was conducted to assess the interactive effects of the packaging film type and storage period on the quality properties and packaging stability of DRK. Pearson correlation analysis was performed, and a correlation matrix plot was generated using R (version 4.3.0, The R Foundation for Statistical Computing, Vienna, Austria) to determine the quality indices used to develop kinetic models for predicting the quality of packaged DRK during storage.

## 3 Results and discussion

### 3.1 Changes in the headspace gas composition and PVE ratio in packaging film pouches

**Fig. 2:**
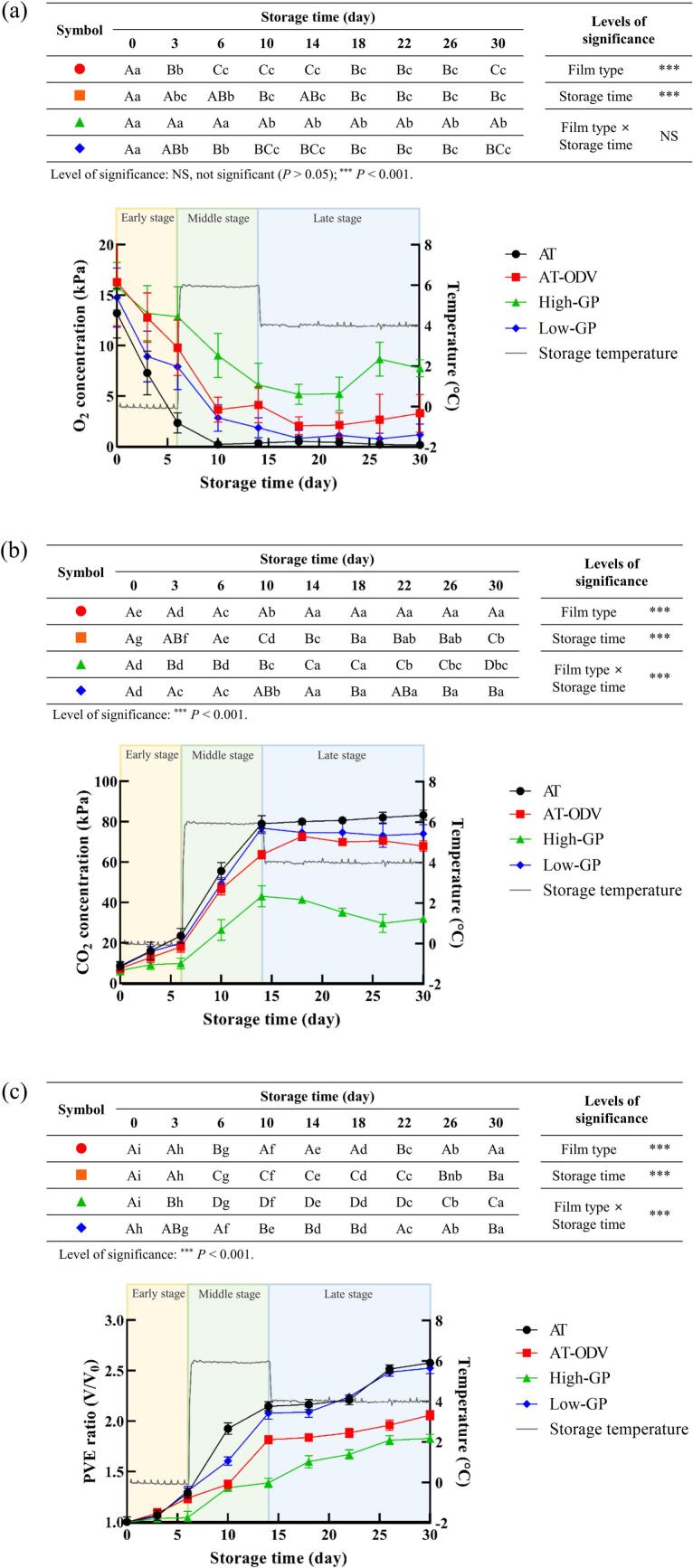
Changes in the headspace O_2_ concentration (a), headspace CO_2_ concentration (b), and PVE ratio (c) of DRK under simulated distribution conditions. Mean values highlighted with different uppercase letters (A–D) differ significantly (*P* < 0.05) under the same storage time. Mean values highlighted with different lowercase letters (a–i) differ significantly (*P* < 0.05) under the same treatment/storage conditions. AT, airtight three-layer; AT-ODV, airtight three-layer film bag equipped with a one-way degassing valve; GP, gas permeable; PVE, packaging volume expansion.

Changes in the headspace O_2_ and CO_2_ concentrations of the AT, AT-ODV, high-GP, and low-GP film bags containing DRK during 30 days of storage under fluctuating temperature conditions are shown in Fig. 2, respectively. Before storage, the headspace O_2_ concentrations were 13.2–16.3 kPa with no significant differences (*P* > 0.05) among the AT (control), AT-ODV, high-GP, and low-GP treatments (Fig. 2a). After the early stage of storage, the headspace O_2_ concentrations in the control were 0.2–0.5 kPa, creating an anaerobic environment. During the middle and late stages of storage, the headspace O_2_ concentrations in the high-GP treatment were higher than those in the AT-ODV and low-GP treatments.

The headspace CO_2_ concentrations in the control and low-GP treatment increased from 8.1 and 8.5 kPa to 79.0 and 76.8 kPa during the early and middle stages of storage, respectively (Fig. 2b). Meanwhile, the lowest headspace CO_2_ concentration of <43.1 kPa was observed in the high-GP treatment during the middle and late stages of storage. The oxygen transmission rate of the high-GP film pouch in this study was 13.5 mL O_2_/m^2^·day·atm, which was lower than that (approximately 10,000 or 20,000 mL O_2_/m^2^·day·atm) of the breathable film pouches reported in previous studies (Park et al., 2022). Nevertheless, no significant packaging swelling was observed in the high-GP film pouch containing DRK during 30 days of storage, maintaining headspace CO_2_ concentrations at 6.3–43.1 kPa. CO_2_ is a primary gaseous byproduct of kimchi fermentation, predominantly generated by heterofermentative LAB species (Lee et al., 2021). The observed changes in headspace gas composition likely reflect a complex interplay between the permeability characteristics of the packaging film and the temperature-driven metabolic activity of the specific LAB counts present. Consequently, the high-GP film effectively mitigated packaging swelling by maintaining a controlled atmosphere with moderate CO_2_ levels.

The PVE ratio in the control increased with prolonged storage period and exceeded 2 during the middle stage of storage (Fig. 2c). In the low-GP treatment, the PVE ratio was 2.5 during the late stage of storage, which led to the expansion of packaging. This result is related to the undesirable deformation of the DRK packaging under fluctuating storage temperature conditions, which can significantly decrease the marketability of the product. This observed packaging expansion is a direct consequence of the excessive accumulation of CO_2_ produced during fermentation within a packaging material that is either impermeable or insufficiently permeable to CO_2_. Although commercial kimchi products are usually not overripe during their distribution period of approximately 25–30 days, products with packaging volumes that have increased by more than two-fold are typically discarded because of consumer rejection (Jaisan et al., 2019). Thus, the PVE ratio can serve as a critical proxy indicator for the effectiveness of gas management within the package and, by extension, the overall quality and marketability of the fermented kimchi.

Conversely, the PVE ratios in the high-GP treatment were maintained at 1.4–1.8 during the middle and late storage periods, and they were significantly (*P* < 0.05) lower than those in the AT-ODV treatment. The superior gas management performance of the high-GP film is attributable to its unique structural design, specifically its four-layered composition that incorporates a micro- and nano-foamed structure (MNFS-PE film) in its interior. This MNFS-PE film is characterized by a large number of micro- or nano-scale pores and channels that are engineered to facilitate gas passage without the risk of liquid leakage (Fig. S1). The effective release of CO_2_ accumulated due to DRK fermentation is crucially dependent on the size of the MNFS-PE film application area. Two-way ANOVA revealed that the type of packaging film bag and storage time exerted interactive effects on the headspace O_2_/CO_2_ concentration and PVE ratio of the film bags containing DRK (*P* < 0.01 or *P* < 0.001). These results indicate the remarkable impact of the high-GP film bag on delaying O_2_ depletion or CO_2_ accumulation in the headspace of DRK packaging. Four-layered GP films with micro- and nano-foamed structures offer a significant advantage in terms of large-scale producibility through a roll-to-roll process while exhibiting promising potential in addressing cost-effectiveness concerns. Therefore, the ability of the high-GP film to maintain low PVE ratios directly translates to an extended shelf life and improved consumer appeal, addressing a major challenge inherent in the packaging of fermented foods.

### 3.2 Changes in TLAB and TC counts

**Fig. 3:**
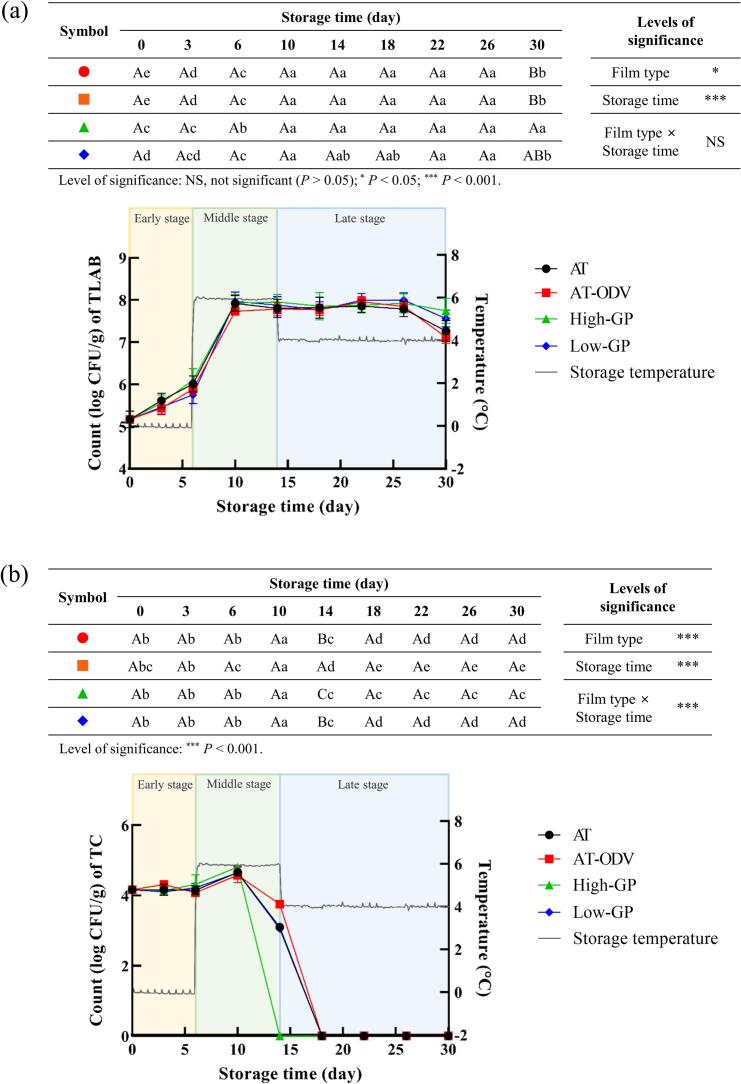
Changes in the counts of TLAB (a) and TC (b) in DRK under simulated distribution conditions. Mean values highlighted with different uppercase letters (A–C) differ significantly (*P* < 0.05) under the same storage time. Mean values highlighted with different lowercase letters (a–e) differ significantly (*P* < 0.05) under the same treatment/storage conditions. AT, airtight three-layer; AT-ODV, airtight three-layer film bag equipped with a one-way degassing valve; GP, gas permeable; TC, total coliforms; TLAB, total lactic acid bacteria.

During the storage and distribution of commercial kimchi, LAB are involved in fermentation, while TC indicates the level of microbiological hygiene. Fig. 3 illustrates the changes in the TLAB and TC counts of the packaged DRK samples during storage under fluctuating temperature conditions. The initial TLAB count in the DRK sample was 4.9 log CFU/g, which was similar to that in the unripened radish kimchi reported by Kim et al. (2018). In the present study, the TLAB count in the control increased to 7.9 log CFU/g during the early and middle stages of storage (Fig. 3a). Similarly, the TLAB counts in the AT-ODV, high-GP, and low-GP treatments increased to 7.8–8.0 log CFU/g, indicating no significant (*P* > 0.05) differences among packaging types. At the end of storage, the TLAB counts in the control, AT-ODV, high-GP, and low-GP treatments ranged from 7.1 log CFU/g to 7.7 log CFU/g (Fig. 3a). During the initial stage of kimchi storage at low temperatures (4–10 °C), aerobic bacteria consume oxygen inside the packaging, thereby fostering an environment suitable for the growth of facultative anaerobes such as *Leuconostoc mesenteroides* (Kim et al., 2012). The decreasing trend in TLAB counts can be ascribed to acid stress caused by organic acid accumulation and nutrient depletion during the late stage of storage. The results of the present study indicated that the growth of TLAB in the DRK samples was affected more by storage temperature than by the packaging used. These results are in agreement with those of a previous report indicating that storage or distribution temperature has a greater influence on the TLAB growth of kimchi than salinity, pH, and packaging type (Di Cagno et al., 2013).

Fig. 3b shows the changes in the TC count of the packaged DRK samples under fluctuating temperature conditions during the storage period. Before and during the early stage of storage, the TC counts were 4.0–4.3 log CFU/g with no significant differences (*P* > 0.05) among the control, AT-ODV, high-GP, and low-GP treatments. The effects of packaging type and storage temperature at 0 °C on TC count remained unaffected. During the middle stage of storage, the TC counts in the control, AT-ODV, high-GP, and low-GP treatments decreased below the detection limit (1 log CFU/g). The TC counts at the detection limit for all the treatments were maintained during the late stage of storage. Song et al. (2019) found that commercial kimchi manufactured with cabbage, radish, or green onions without heat sterilization has an initial TC count of 2.1–5.2 CFU/g before fermentation, which is similar to the findings of the present study. However, during the ripening stage of kimchi, organic acids produced during lactic acid fermentation pass through the cell membranes of coliforms, decreasing the intracellular pH and damaging cell proteins and DNA structures. This ultimately renders coliforms inactive. Consistent with our results, coliform inactivation has also been observed in young radish and leaf mustard kimchi samples fermented to a TA of approximately 6–7 g/L (Lee et al., 2021; Yun et al., 2024). Therefore, the maturation process of kimchi must be managed to eliminate TC and guarantee the microbiological safety of the product.

### 3.3 Changes in pH, TA, RS content, and salinity

**Fig. 4:**
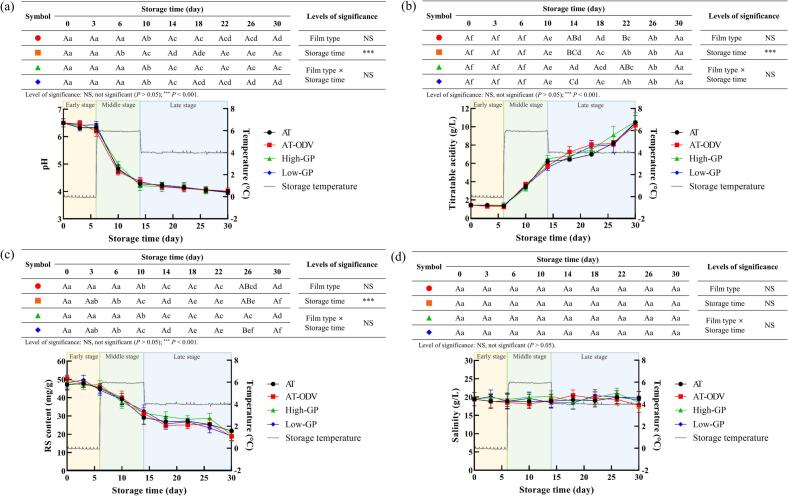
Changes in the pH (a), TA (b), RS content (c), and salinity (d) of DRK under simulated distribution conditions. Mean values highlighted with different uppercase letters (A–C) differ significantly (*P* < 0.05) under the same storage time. Mean values highlighted with different lowercase letters (a–f) differ significantly (*P* < 0.05) under the same treatment/storage conditions. AT, airtight three-layer; AT-ODV, airtight three-layer film bag equipped with a one-way degassing valve; GP, gas permeable; RS, reducing sugar; TA, titratable acidity.

Kimchi fermentation by LAB leads to the production of organic acids from sugars, which alter the pH and acidity of kimchi (Baek et al., 2020). Fig. 4a shows the changes in the pH of the packaged DRK samples during storage under fluctuating temperature conditions. The initial pH of DRK was 6.5. During the early stage of storage, the pH remained at 6.2–6.4 in the control, AT, AT-ODV, high-GP, and low-GP treatments. During the middle stage of storage, the pH in the control and all treatments decreased to 4.2–4.4, indicating an optimal ripening status. These results indicated that regardless of the packaging used, all DRK samples had a pH of 4.0 by the end of storage.

Contrary to the pH results, the TA values significantly increased (*P* < 0.05) from 1.4 g/L to 5.5–6.5 g/L in all four treatments during the early and middle stages of storage (Fig. 4b). The TA values in the control, AT, AT-ODV, high-GP, and low-GP treatments were 10.2–10.6 g/L after 30 days of storage. These results indicated that the type of packaging film did not affect the acidity of DRK. Similarly, a previous study reported that the pH and TA of kimchi at the optimal ripening stage are 4.2–4.5 and 5–9 g/L, respectively (Park et al., 2022). Therefore, regardless of the packaging used, the optimal ripening state of the DRK samples was maintained between 14 and 26 days of storage.

During the early stage of storage, the RS contents of DRK were 47.1–50.2 mg/g with no significant differences among the AT, AT-ODV, high-GP, and low-GP film treatments (Fig. 4c). Thereafter, the RS contents decreased by 31.7 and 32.9 mg/g in the high-GP and low-GP treatments, respectively, during the middle stage of storage, but these values were not significantly different from those obtained in the control and AT-ODV treatments (*P* > 0.05). At the end of storage, the RS contents in all treatments were 18.9–21.8 mg/g, representing approximately 40 % of the initial RS content before storage. The main RSs in radish kimchi are glucose, fructose, and galactose (Choi et al., 2023). The reduction in RS levels of the DRK samples can be ascribed to the RS consumption of LAB during fermentation (Jung et al., 2012). Two-way ANOVA further confirmed that packaging type had no significant effect (*P* > 0.05) on the RS content of DRK.

The salinity of the control, AT-ODV, high-GP, and low-GP treatments was 18.4–21.1 g/L before and during storage for 30 days (Fig. 4d). Two-way ANOVA confirmed that the DRK salinity was not significantly influenced (*P* > 0.001 or *P* > 0.05) by packaging types and their interaction with the storage period. These results were similar to those of a previous study (Lee et al., 2019) indicating that the salinity of Korean cabbage kimchi does not significantly change (*P* > 0.05) before and after storage at 4 and 10 °C for 6 weeks.

Choi et al. (2023) reported that the size of radish cube can significantly affect the pH, acidity, and metabolite production during radish kimchi fermentation. However, a limitation of the present study is that the effects of different packaging film bags on the physicochemical characteristics of radish kimchi manufactured with radish cubes of different sizes were not evaluated.

### 3.4 Correlation between microbiological and chemical quality characteristics of DRK, packaging deformation index, and storage time

**Fig. 5:**
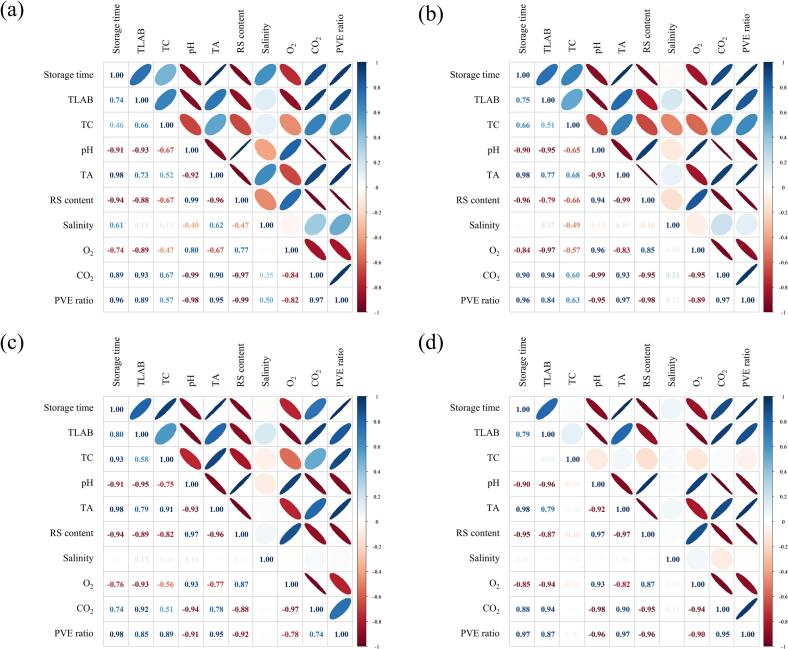
Pearson correlation analysis of variables of DRK packaged in AT (a), AT-ODV (b), high-GP (c), and low-GP (d) under simulated distribution conditions. AT, airtight three-layer; AT-ODV, airtight three-layer film bag equipped with a one-way degassing valve; GP, gas permeable; DRK, diced radish kimchi.

Fig. 5 displays the Pearson correlation results among the TLAB count, pH, TA, RS content, salinity, headspace gas concentration, PVE ratio, and storage time. A Pearson correlation coefficient approaching +1 or −1 signifies a strong positive or negative linear relationship, respectively (Sadat, 2024). In all treatments, storage time showed a significant positive correlation (*P* < 0.05) with TA and PVE ratio, with correlation coefficients of 0.96–0.98. The relationship between the CO_2_ concentration in the packaging headspace and storage time was influenced by the type of packaging film, as evidenced by the correlation coefficients ranging between 0.74 and 0.90. By contrast, storage time had a significant negative correlation (*P* < 0.05) with pH and RS content. No significant correlation (*P* > 0.05) was found between salinity and storage time or other chemical quality parameters for any packaging type, indicating that the metabolites produced by DRK fermentation did not affect salinity. When using chemical quality indicators to determine the maturity of kimchi, TA, which has a consistent change pattern, is more accurate than pH because the latter is affected by buffering capacity during storage (Jaisan & Lee, 2017). Establishing a specific threshold value as a validated quality indicator is difficult because the initial RS content of kimchi varies depending on the content of its constituent ingredients. Lee et al. (2019) found that the change in TA during storage is a crucial quality indicator for estimating the shelf life of cut cabbage kimchi by using regression analysis models. Based on the above-mentioned results, TA and PVE ratio were chosen as the dependent variables to develop kinetic models for predicting the quality and packaging stability of packaged DRK during storage.

### 3.5 Kinetic modeling for the prediction of TA and PVE ratio changes during storage

Table 2, Table 3 summarize the kinetic parameters for the TA and PVE ratios of DRK packaged in different film bags under fluctuating storage temperatures, which were obtained by fitting the linear, Gompertz, logistic, and Huang models, along with their corresponding goodness-of-fit parameters. In all packaging treatments, the logistic models presented lower *μ*_*max*_ values of TA than the Gompertz and Huang models (Table 2). The *λ* values calculated from the Huang model fitting TA data were 4.02–4.74 d in the control, AT-ODV, high-GP, and low-GP treatments. These findings indicated that the onset of TA increase, detectable by the initiation of DRK fermentation, was delayed during the early stage of storage. Furthermore, fitting the four kinetic models using the PVE ratio data revealed remarkable differences in *μ*_*max*_ values, which were attributed to the type of packaging film used for DRK (Table 3). The *λ* values of the PVE ratio in the high-GP treatment were higher than those in the control, AT-ODV, and low-GP treatments (Table 3). Therefore, the *μ*_*max*_ and *λ* parameters are pivotal for understanding the DRK fermentation kinetics and potentially optimizing packaging methods and storage conditions.

**Table 2:** Kinetic and goodness-of-fit parameters of mathematical models for TA prediction of diced radish kimchi under fluctuating temperature storage conditions.

Parameter	Treatment	Model	Kinetic parameter		Goodness-of-fit parameter				
			*μ*_*max*_ (g/L·d^−1^)	*λ* (d)	*R*^*2*^_*adj*_	*RMSE*	*AICc*	*A*_*f*_	*B*_*f*_
TA	AT (control)	Linear	–	–	0.92	0.84	**−**0.57	1.12	1.01
		Gompertz	0.33	3.39	0.94	0.72	**−**3.34	1.08	1.04
		Logistic	0.19	5.07	0.93	0.75	**−**2.61	1.07	0.99
		Huang	0.30	4.08	0.96	0.61	**−**6.37	1.05	1.01
	AT-ODV	Linear	–	–	0.94	0.71	**−**3.59	1.11	1.00
		Gompertz	0.39	2.47	0.95	0.66	**−**4.91	1.07	1.06
		Logistic	0.14	6.03	0.95	0.64	**−**5.46	1.08	1.00
		Huang	0.34	4.74	0.96	0.58	**−**7.38	1.04	1.02
	High-GP	Linear	–	–	0.93	0.81	**−**1.22	1.12	1.00
		Gompertz	0.38	2.54	0.96	0.66	**−**4.91	1.07	0.98
		Logistic	0.14	5.96	0.95	0.72	**−**3.34	1.06	0.98
		Huang	0.37	4.07	0.97	0.58	**−**7.37	1.04	1.01
	Low-GP	Linear	–	–	0.94	0.75	**−**2.61	1.11	0.97
		Gompertz	0.35	2.82	0.95	0.68	**−**4.37	1.07	1.05
		Logistic	0.14	6.19	0.95	0.63	**−**5.75	1.08	0.97
		Huang	0.36	4.02	0.97	0.48	**−**10.58	1.04	1.01

TA, titratable acidity; AT, airtight three-layer; AT-ODV, airtight three-layer film bag equipped with a one-way degassing valve; GP, gas permeable.

**Table 3:** Kinetic and goodness-of-fit parameters of mathematical models for PVE ratio prediction of diced radish kimchi under fluctuating temperature storage conditions.

Parameter	Treatment	Model	Kinetic parameter		Goodness-of-fit parameter				
			*μ*_*max*_ (d^−1^)	*λ* (d)	*R*^*2*^_*adj*_	*RMSE*	*AICc*	*A*_*f*_	*B*_*f*_
PVE ratio	AT (control)	Linear	–	–	0.90	0.17	**−**29.39	1.04	1.00
		Gompertz	0.10	1.99	0.95	0.12	**−**35.59	1.02	0.99
		Logistic	0.11	2.18	0.95	0.12	**−**35.87	1.02	1.00
		Huang	0.16	2.59	0.97	0.10	**−**39.00	1.02	1.00
	AT-ODV	Linear	–	–	0.89	0.12	**−**35.67	1.03	1.01
		Gompertz	0.07	2.95	0.95	0.08	**−**42.89	1.02	0.99
		Logistic	0.08	2.61	0.94	0.09	**−**40.97	1.02	1.02
		Huang	0.11	2.70	0.97	0.06	**−**47.39	1.01	1.00
	High-GP	Linear	–	–	0.89	0.10	**−**39.60	1.02	1.01
		Gompertz	0.05	3.44	0.94	0.07	**−**45.30	1.02	1.02
		Logistic	0.04	3.84	0.93	0.08	**−**42.89	1.02	1.00
		Huang	0.06	3.31	0.97	0.05	**−**49.88	1.02	1.00
	Low-GP	Linear	–	–	0.92	0.15	**−**31.98	1.03	0.99
		Gompertz	0.09	2.06	0.98	0.07	**−**45.30	1.01	1.00
		Logistic	0.09	1.87	0.98	0.08	**−**43.09	1.02	1.00
		Huang	0.12	2.52	0.99	0.06	**−**47.02	1.01	1.00

PVE, packaging volume expansion; AT, airtight three-layer; AT-ODV, airtight three-layer film bag equipped with a one-way degassing valve; GP, gas permeable.

*R*^*2*^_*adj*_ is used to assess the fit of a model, indicating the percentage of variance explained by the model. An *R*^*2*^_*adj*_ value close to 1 suggests a high level of agreement between the observed and predicted values (Botella et al., 2023). *RMSE* values are obtained by comparing the predicted values of the models with the observed values in the experiment. The lower the *RMSE* value, the more reliable and accurate the model (Baranyi et al., 1996). In addition, *AICc* is an estimator of the relative quality of statistical models for a given set of data (Abidi et al., 2022). A model with a lower *AICc* value can accurately predict the dependent variable using fewer parameters (McGregor et al., 2000). For the linear, Gompertz, logistic, and Huang models fitted using the TA increase data in the context of the control, AT-ODV, high-GP, and low-GP treatments stored at fluctuating temperatures, the *R*^*2*^_*adj*_ values were closer to 1 (≥ 0.93), whereas the *RMSE* and *AICc* values were lower than 0.75 and − 2.61, respectively (Table 2). Among the four mathematical models fitted to the PVE ratio data, higher *R*^*2*^_*adj*_ and lower *RMSE* and *AICc* values  were observed in the Huang model than in the linear, Gompertz, and logistic models (Table 3).

The closer the absolute values of *A*_*f*_ and *B*_*f*_ are to 1, the better is the match between the observed and predicted values (Menezes et al., 2018). The *A*_*f*_ and *B*_*f*_ components of the Huang model fitted using the TA and PVE ratio increase data were between 1.00 and 1.05, which are acceptable ranges with respect to the conformity between the experimental and predicted data (Table 2, Table 3). These results indicated that the Huang model showed acceptable performance in predicting the changes in the TA and PVE ratio of the packaged DRK samples stored under limited fluctuating temperature conditions.

These results indicate that combining high-GP film packaging with quality prediction models is a potential strategy to maintain marketability and prevent disposal issues during the storage and distribution of DRK. Further research is needed to ensure that the developed models can be popularized for practical applications in the kimchi industry.

## 4 Conclusions

DRK was packaged using a high-GP four-layered film bag laminated with a functional LDPE film featuring micro- and nano-foamed structures. This novel innovative design effectively suppressed CO_2_ accumulation in the headspace of the packaging during storage at fluctuating temperatures while preventing volume expansion. Unlike the fluctuating storage temperature conditions, the gas permeability of the packaging film bag and the attachment of a one-way degassing valve did not significantly affect (*P* > 0.05) the TLAB count, pH, TA, and RS content of DRK during storage. Additionally, kinetic models were constructed to predict the changes in the TA and PVE ratio of DRK in different packaging film bags during storage. The Huang model fitted using TA or PVE ratio data demonstrated higher *R*^*2*^_*adj*_ values or lower *RMSE*, *AICc*, *A*_*f*_, and *B*_*f*_ values than the linear, Gompertz, and logistic models, making it suitable for estimating the primary shelf life of DRK. Our findings may serve as a reference for producers and distributors in devising stock strategies and managing the quality of kimchi products and for consumers in determining the optimal time for consumption. Further studies are needed to verify the reliability of the TA and PVE ratio predictive models developed in this study under practical storage and distribution conditions.

## CRediT authorship contribution statement

**So Yoon Park:** Writing – original draft, Visualization, Resources, Methodology, Formal analysis. **Suk-Min Yun:** Software, Formal analysis. **Jeong-Yong Cho:** Validation, Data curation. **Bo-Sung Shin:** Validation, Methodology. **Ho Hyun Chun:** Writing – review & editing, Supervision, Project administration, Conceptualization.

## Declaration of competing interest

The authors declare that they have no known competing financial interests or personal relationships that could have appeared to influence the work reported in this paper.

## Data Availability

Data will be made available on request.

## Appendix A Supplementary data

Supplementary material. Image 1
