# Supplementary material for: Application of innovative gas-permeable four-layered film for packaging diced radish (Raphanus sativus L.) kimchi to extend shelf life under fluctuating temperature conditions
Source: Food Chem X. 2025 Sep 8;30:103008. doi: 10.1016/j.fochx.2025.103008 (PMC12455112; doi:10.1016/j.fochx.2025.103008)
Supplement: Supplementary file 1 — Supplementary material. [file mmc1.docx]

Supporting Information

**Application of innovative gas-permeable four-layered film for packaging of diced radish (*Raphanus sativus* L.) kimchi to extend shelf life under fluctuating temperature conditions**

So Yoon Park^a,b^, Suk**-**Min Yun^a,b^, Jeong**-**Yong Cho^b^, Bo-Sung Shin^c^, Ho Hyun Chun^a,*^

^a^*Industrial Intelligence Research Division, World Institute of Kimchi, Gwangju 61755, Republic* *of Korea*

^b^*Department of Integrative Food, Bioscience and Biotechnology, Graduate School of Chonnam National University, Gwangju* *61186, Republic of Korea*

^c^*Department of Optics and Mechatronics Engineering, Pusan National University, Busan 46241, Republic of Korea*

^*^Corresponding author.

*E-mail address:* hhchun@wikim.re.kr (H. H. Chun).


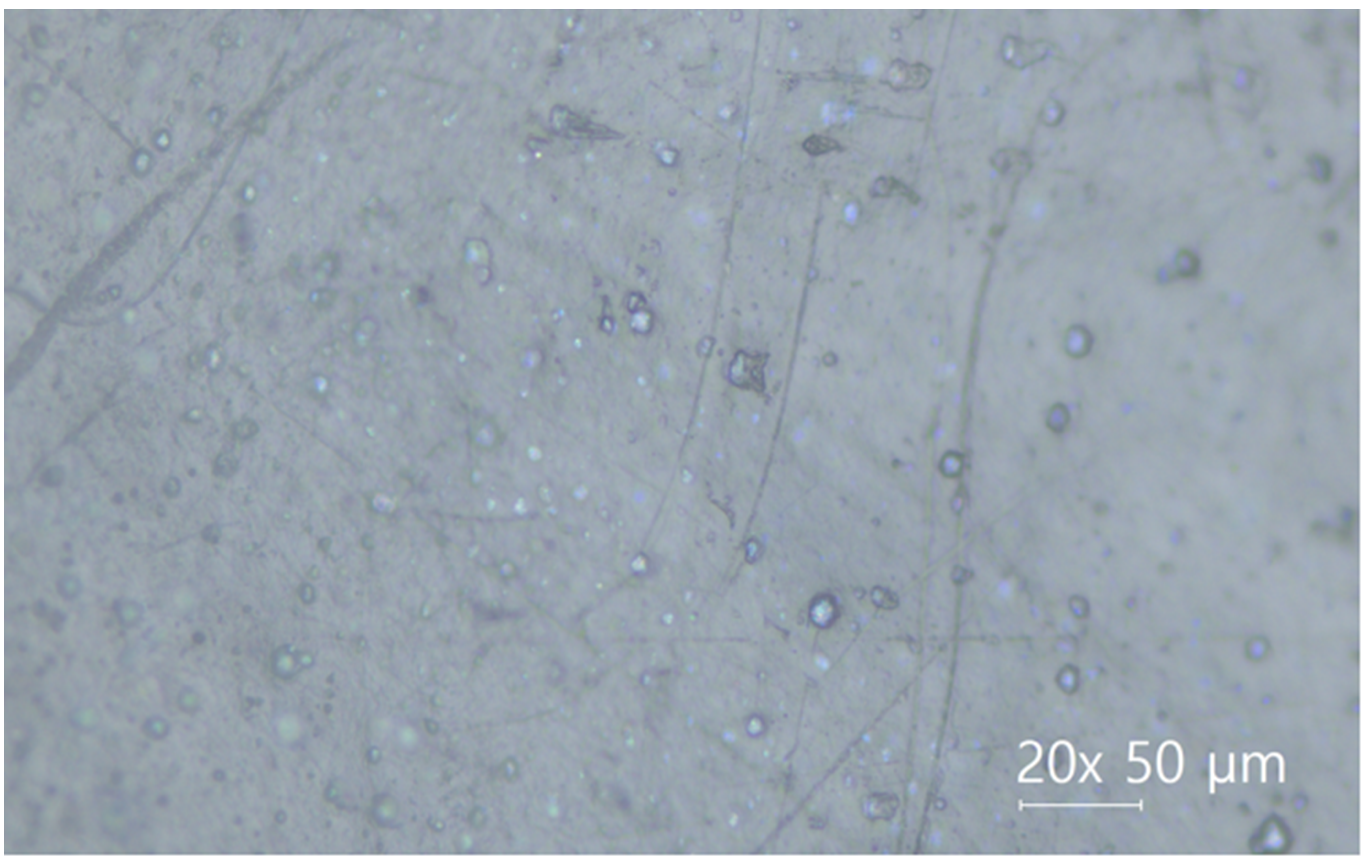


**Fig. S1.** Optical micrograph of micro- or nano-scale pores and channels in MNFS-PE film.
